# Shift Work as a Potential Risk Factor for Lower Ovarian Reserve: A Study of Fertility Patients

**DOI:** 10.3390/jcm15124769

**Published:** 2026-06-19

**Authors:** Adeolu Banjoko, Nina Harris, Sara Mousavi, Stella Wang, Ella Huszti, Zachary M. Ferraro, Claire Ann Jones

**Affiliations:** 1 Department of Obstetrics and Gynaecology, Temerty Faculty of Medicine, University of Toronto, 1 King’s College Circle, Medical Sciences Building Room 2109, Toronto, ON M5S 1A8, Canada; nina.harris@dal.ca (N.H.); sara.mousavi@mail.utoronto.ca (S.M.); claire.jones@mail.utoronto.ca (C.A.J.); 2 Department of Biostatistics, University Health Network, Toronto, ON M5G 1X8, Canada; stella.wang@thebru.ca (S.W.);; 3 Mount Sinai Fertility, Sinai Health System, 250 Dundas Street West Suite 700, Toronto, ON M7A 1G1, Canada

**Keywords:** ovarian reserve, circadian rhythm, shift work, fertility, anti-Müllerian hormone

## Abstract

**Background/Objectives:** Shift work is a form of circadian dysregulation, which has been associated with adverse reproductive health outcomes. However, the association between circadian dysregulation and ovarian reserve remains uncertain. The present study examines whether shift work is associated with lower AMH levels in women seeking fertility treatment. **Methods:** This retrospective cohort study includes female patients aged 20–39 years presenting between February 2023 and June 2024. Patients were excluded if they had only one ovary, a current cancer diagnosis, or past chemotherapy use. Demographic and medical data were obtained from the electronic medical record. AMH levels were compared between daytime workers and shift workers. **Results:** A total of 1135 patients met inclusion criteria. The median age was 35 years (IQR 32–37). Of these, 89% (*n* = 1014) reported daytime work, and 11% (*n* = 121) reported shift work, comprising 102 working rotating shifts, seven working night shifts, and 12 working evening shifts. Daytime-only workers had a median AMH of 17.20 pmol/L (9.1–30.0). Combined shift workers had a median AMH of 17.10 pmol/L (8.1–31.0). There was no statistically significant difference in AMH levels between daytime workers and shift workers (*p* = 0.935). Although not significant, the odds of having low AMH levels (<7 pmol/L) were 25% higher among shift workers compared to daytime workers (OR 1.246, *p* = 0.345). **Conclusions:** In this cohort, AMH levels did not significantly differ between daytime and shift workers, offering reassurance to individuals required to engage in shift work. Future research should include larger cohorts and incorporate more comprehensive measures of circadian disruption.

## 1. Introduction

Circadian rhythms follow a 24 h cycle that links environmental cues to physiological processes and subsequent health outcomes [1]. The suprachiasmatic nucleus in the hypothalamus regulates peripheral CLOCK genes, including those involved in reproduction [2]. The molecular clock functions through transcription–translation feedback loops and thereby alters physiology and behaviours in response to outside environmental cues [2]. Shift work has been demonstrated to lead to circadian disruption and adverse health outcomes [3].

Within the ovary, granulosa cells, theca cells and oocytes exhibit genes that oscillate within the circadian cycle [2], and hormone production critical for female reproductive function is influenced by circadian rhythms [2]. Studies have demonstrated that sex-steroid hormones, gonadotropins, and sex hormone binding globulin (SHBG) are all under endogenous circadian control [1,2,4]. Circadian clock gene expression in granulosa cells declines with physiologic ageing, suggesting an association between circadian regulation and ovarian ageing [5].

Diminished ovarian reserve (DOR) affects approximately 26% of women presenting for infertility evaluation and is associated with lower pregnancy rates, poorer ovarian stimulation response, and increased IVF cycles needed to achieve pregnancy [6,7,8]. Clinically, ovarian reserve assessment is essential for treatment planning and patient counselling and is commonly based on antral follicle count (AFC) and anti-Müllerian hormone (AMH) levels [9,10]. AMH is used for individualized gonadotropin dosing and to predict poor ovarian response in IVF cycles, with DOR often defined as AMH < 0.5–1.1 ng/mL (3.6–7.9 pmol/L) [11,12].

The relationship between circadian disruption and ovarian reserve has only recently been explored. Mínguez-Alarcón et al. first reported lower mature oocyte yields among shift workers undergoing ovarian stimulation, although AMH was not assessed [13]. Johnson et al. found no association between shift work and AMH in a healthy population [14]. More recently, Lin et al. reported that poor sleep quality was associated with lower AMH and AFC, potentially supporting a link between circadian rhythms and ovarian reserve [15].

Circadian misalignment has been associated with irregular menstrual cycles, altered reproductive hormone levels, prolonged time to conception, and increased risk of spontaneous abortion [16]. However, the role of circadian misalignment, such as that experienced during shift work, on ovarian reserve remains poorly understood. A decline in ovarian reserve has a multifactorial etiology, and identifying modifiable risk factors like circadian disruption could offer new insights into its pathophysiology [10]. With the global rise in infertility and increasing prevalence of circadian disruption due to modern lifestyles and shift work, understanding this relationship is essential and timely [8].

The present study aims to evaluate whether shift work is associated with lower AMH levels compared to daytime work in women presenting to a fertility clinic.

## 2. Materials and Methods

### 2.1. Study Design

This is a retrospective cohort study performed at Mount Sinai Fertility in Toronto, Canada.

### 2.2. Ethical Approval

Research Ethics Board Approval for retrospective chart review was obtained from Mount Sinai Hospital for this study (REB #23-0029-C).

### 2.3. Study Population

The population includes patients with a female health card aged 20–39 years presenting to Mount Sinai Fertility for initial consultation from 1 February 2023 to 30 June 2024. Patients were included if information on work hours was documented in the initial consultation note. Patients working shift work were defined as those who self-reported working nights, evenings or rotating shifts and were compared to patients who self-reported daytime-only work. The outcome of interest was serum AMH levels drawn within 6 months of the initial consultation appointment. AMH levels drawn at the Mount Sinai Fertility laboratory used the UniCel DxI 600 automated analyser assay manufactured by Beckman Coulter, Chaska, MN, USA, sourced through Beckman Coulter Canada, LP in Missisauga, ON, Canada.

Exclusion criteria were patients with one ovary, no AMH level taken during the study period, a current diagnosis of cancer, previous use of chemotherapeutic agents, or if there was no data regarding their working hours recorded in the electronic medical record.

### 2.4. Data Collection

A data query in the clinic’s electronic medical record (eIVF) for patients presenting for initial consultation from 1 February 2023 to 30 June 2024 was performed to identify potential patients to be included in the study. Data was collected by review of the electronic medical record.

A prompt to record work hours was added to the initial consultation note template for all new patients from February 2023 onwards with drop-down options including “day shifts only”, “evening shifts only”, “night shifts only” or “rotating shifts”. The menu of options to document patient responses was modelled on the Statistics Canada Canadian Health Measures Survey (CHMS) question which assessed shift work [17].

Serum AMH levels were drawn either on site or at external labs according to patient preference with results populated into the electronic medical record. Serum AMH levels, including units and the date of the AMH test, were extracted from the electronic patient health record (eIVF) for this study.

Demographical data such as age, ethnicity, occupation, and body mass index (BMI) were recorded. Gravidity and parity, pre-existing medical conditions including the presence of polycystic ovary syndrome (PCOS) or endometriosis, gynecological surgery history, and medication use including hormonal medications were collected, as well as use of tobacco or cannabis. Partner information was recorded, including if the patient had a partner and the working hours of the partner.

### 2.5. Statistical Analysis

For statistical analysis, patients were categorized into cohorts according to work schedule, daytime work or shift work, with the latter further classified into rotating, evening, and night shifts. Patient characteristics were reported as median (interquartile range, IQR) for continuous variables, and frequency (percentage) for categorical variables, and were compared between daytime workers and shift workers using Mann–Whitney Test and chi-squared test or Fisher’s exact test, as appropriate.

Group-specific medians (IQR) for AMH were reported overall and within the shift work subcategories. To evaluate differences in AMH between cohorts, univariable and multivariable linear regression were fitted with log-transformed AMH levels as the outcome. Regression coefficients were back-transformed and expressed as percentage differences. Multivariable linear regressions were adjusted for variables demonstrating imbalance or considered clinically relevant, including use of hormonal medications, the presence of PCOS, age and BMI. The likelihood of DOR was also evaluated using univariable and multivariable logistic regression. The multivariable models were adjusted for the same potential confounders.

Because of the heterogeneity of AMH levels among patients with PCOS, a sensitivity analysis excluding PCOS patients was performed to improve comparability with prior studies and potentially increase statistical power [14,15].

All statistical analyses were performed in R (version 4.2.1). A two-sided *p*-value < 0.05 was considered statistically significant.

## 3. Results

A total of 3535 patient charts were reviewed, with 1135 participants meeting the inclusion criteria (Table 1).

Of these 1135 included participants, 89% (*n* = 1014) worked daytime hours, and 11% (*n* = 121) performed shift work. Among shift workers, 102 worked rotating shifts, seven worked night shifts only, and 12 worked evening shifts only.

The median age across the study was 35 [32.00, 37.00] years old. The median BMI was 24.10 kg/m^2^ [21.44–28.20] (Table 1). Baseline characteristics were similar between groups except for a significantly higher proportion of patients using hormonal medications among shift workers compared with daytime workers (9.1% vs. 2.6%, *p* < 0.001). Occupations categorized using Canadian National Occupational Classifications (NOCs) differed, with healthcare predominating among shift workers and professional/managerial roles among daytime workers.

Table 2 demonstrates AMH levels by working hours. AMH levels varied widely, with a median [IQR] of 17.20 pmol/L [9.0–30.0] for all patients. Daytime workers had a median AMH of 17.20 pmol/L [9.1–30.0]. The median AMH for combined shift workers was 17.10 pmol/L [8.1–31.0]. Among shift workers, the median AMH for rotating shift workers was 17.05 pmol/L [8.1–30.8], night shift workers 23.00 pmol/L ([3.3–23.3], and evening shift workers 20.12 pmol/L ([0.5–41.0].

For regression coefficients, AMH was log-transformed due to its non-normal distribution; coefficients were back-transformed and are expressed as percentage differences. In univariable and multivariable models accounting for hormonal medication use, PCOS, age and BMI, shift work was not associated with AMH levels with estimates close to zero and wide adjusted confidence intervals (β = −0.85%, *p* = 0.935; β = 2.30%, *p* = 0.823) (Table 3).

Age was strongly associated with lower AMH levels (β = −6.67%, *p* < 0.001), while PCOS was associated with higher AMH levels (β = 155.03%, *p* < 0.001). Underweight and obese BMI categories were associated with lower AMH compared with normal weight (β = −37.75%; *p* = 0.003; β = −17.34%; *p* = 0.026). Use of hormonal medications was not significantly associated with AMH levels (β = −24.05%, *p* = 0.122).

A sensitivity analysis excluding PCOS patients, due to the heterogeneity of AMH levels in these patients, also showed no significant association between shift work and AMH (β = −2.43%, *p* = 0.821).

We found 27 (22.3%) of the shift working patients had a low AMH (<7 pmol/L), compared to 190 (18.7%) of the patients who work daytime hours (Table 2). In logistic regression models, shift work was not associated with low AMH (<7 pmol/L). In univariable logistic regression (Table 4a), shift work was associated with a non-significant 24.6% increase in the odds of low AMH compared with daytime work (OR = 1.246; *p* = 0.345). However, history of ovarian surgery was associated with increased odds of low AMH (2.175, *p* = 0.039).

Similarly, after multivariable logistic regression analysis adjusted for hormonal medication use, PCOS, age and BMI (Table 4b), shift work was not associated with low AMH levels (OR = 1.14, *p* = 0.606). Results remained consistent in the sensitivity analysis with PCOS patients excluded (OR = 1.19; *p* = 0.492).

Age (OR = 1.17, *p* = <0.001) and overweight BMI (OR = 1.51, *p* = 0.030) were associated with higher odds of low AMH, while PCOS was associated with lower odds (OR = 0.276; *p* = 0.003). Underweight BMI was associated with low AMH but was not statistically significant (OR 2.02, *p*= 0.058). The association between hormonal medication use and AMH was not significant.

## 4. Discussion

In this retrospective cohort study of 1135 patients undergoing fertility evaluation, we found no statistically significant associations between shift work status and ovarian reserve, measured by AMH levels. Both unadjusted and multivariable regression models consistently showed no significant differences in AMH levels or odds of low AMH (<7 pmol/L) among shift workers compared to those working daytime hours. These findings persisted after excluding patients with PCOS.

Although point estimates suggested slightly lower AMH levels and increased odds of low AMH in shift workers, particularly after exclusion of PCOS patients, these associations were not statistically significant. The proportion of participants engaged in shift work in our cohort was lower than the 26% prevalence reported in Canadian statistical data, which may have reduced statistical power [18].

Our cohort had a high proportion of professional and managerial workers, for whom extended or irregular hours may occur even in nominally daytime schedules. As such, occupational shift classification may inadequately reflect true circadian disruption in this population, potentially attenuating detectable differences in AMH and limiting generalizability to groups with more clearly defined work patterns.

Additionally, AMH is a complex and variable biomarker influenced by multiple factors beyond circadian rhythm. In our analysis age was the strongest predictor of lower AMH levels, reaffirming the prior literature [6]. In addition, PCOS was predictably and strongly associated with elevated AMH levels, and prior ovarian surgery significantly increased the odds of low AMH, highlighting the impact of these potential covariates. In our study, we accounted for these factors in our baseline characteristic analysis, multivariable models, or sensitivity analysis excluding patients with PCOS. Although a history of ovarian surgery was associated with increased odds of low AMH in univariable analysis, this variable was not included in the final multivariable model because there was no significant difference in the prevalence of prior ovarian surgery between shift workers and daytime workers in baseline characteristics. As ovarian surgery was not associated with the exposure of interest (work schedule) in our study, or in the similar prior literature, we deemed it was unlikely to act as a confounder of the relationship between shift work and AMH and therefore was not included in the final adjusted model [13,14,15]. Our study assessed ovarian reserve using AMH alone, a reliable marker of ovarian reserve [11]. However, the inclusion of AFC would have provided more rigorous results and this represents a study limitation.

Our findings highlight the limitations of using shift work as a surrogate for circadian disruption. While night, evening, and rotating shifts contribute to circadian misalignment, they are proxies that may not account for individual variability in sleep–wake patterns, light exposure, or chronotype. Furthermore, shift work may encompass occupational exposures beyond circadian disruption, including differences in physical workload and psychosocial stress [19]. Objective measures such as melatonin or cortisol profiling, sleep studies, or validated sleep questionnaires may better characterize physiologic circadian misalignment [20].

Furthermore, the heterogeneity within the shift work group may have diluted potential effects. Research suggests that the physiological impact of consistent night shifts may be less substantial than rotating shifts due to the potential for partial physiological adaptation [20]. We did not have the sample size to do a separate analysis for each type of shift working group. There was not a uniform definition for “rotating shifts” explained to patients, and thus this sub-cohort was likely a heterogenous group with varying combinations of day, evening, and night shift work of differing intensities. It is worth exploring in future studies whether cumulative exposure to shift work, such as the number of years or frequency of night shifts, as well as the intensity of work, plays a role in ovarian reserve. Prospective studies or studies with linked employment records may offer an opportunity to better quantify the duration and timing of circadian disruption.

Prior studies examining circadian disruption and ovarian reserve have yielded mixed findings. In the EARTH study, shift work was associated with fewer mature oocytes retrieved during IVF, particularly among evening and night shift workers, suggesting that stimulation response may be more sensitive to circadian influences than markers such as AMH [13].

In contrast, Johnson et al. found no association between night shift work and AMH levels among premenopausal nurses in the Nurses’ Health Study II, despite assessing both recent and cumulative shift work exposure, consistent with our findings [14]. More recently, Lin et al. reported that poor sleep quality, assessed using the Pittsburgh Sleep Quality Index, was associated with lower AMH and AFC, while shift work itself was not [15]. Together, these findings suggest that sleep quality and restorative sleep may be more relevant to ovarian reserve than occupational shift timing alone, underscoring the limitations of shift work classification as a surrogate for circadian disruption [20,21]. Optimizing sleep hygiene could thus be an important consideration in counselling shift workers and other patients undergoing fertility assessment.

### Strengths and Limitations

Strengths of our study include the large sample size, multivariable modelling, and sensitivity analyses excluding PCOS.

Limitations include a reliance on self-reported work hours, the lack of more objective circadian or sleep measures, and potential residual confounding by lifestyle or socioeconomic factors. The occupational composition of our cohort, with a high proportion of healthcare workers among the shift workers and professional or managerial workers among the daytime workers, may limit generalisability to broader populations seeking fertility care. These occupations may also be associated with distinct occupational stressors that could confound the relationship between working hours and ovarian reserve, but we did not perform a sensitivity analysis for this potential confounder because healthcare workers comprised the majority of the shift work cohort. Excluding these participants would have substantially reduced the exposed group and further limited statistical power, making the results difficult to interpret. Additionally, professional occupations were also overrepresented in the daytime working cohort. Furthermore, the study is limited by its single-centre design. Future multi-centre studies incorporating measures of sleep quality as a surrogate marker of circadian disruption may help further elucidate the relationship between circadian disruption and ovarian reserve. However, this study provides unique real-world insight into the relationship between work schedules and ovarian reserve markers in patients actively undergoing fertility assessment.

## 5. Conclusions

No significant association was found between shift work and ovarian reserve as measured by AMH levels. These results suggest that shift work, as a proxy for circadian rhythm disruption, may not have a major impact on ovarian reserve detectable via AMH in a fertility clinic population. Given the methodological limitations in circadian exposure measurement, further research with larger samples and more precise circadian assessments is needed. A better understanding of how circadian disruption affects reproductive endocrinology may help inform fertility counselling and occupational health recommendations.

## Figures and Tables

**Table 1:** Baseline characteristics of 1135 participants enrolled in the study.

Characteristics		Overall	Shift Work	Daytime Work	*p*-Value
*n*		1135	121	1014	
Age (years)		35.00 [32.00, 37.00]	35.00 [32.00, 37.00]	35.00 [32.00, 37.00]	0.328
BMI (kg/m^2^)		24.10 [21.44, 28.20]	23.80 [21.28, 27.74]	24.17 [21.48, 28.23]	0.529
BMI Class	Underweight (<18.5)	43 (3.9)	7 (6.1)	36 (3.7)	0.515
	Normal Weight (18.5–24.9)	564 (51.5)	58 (50.9)	506 (51.6)	
	Overweight (25.0–29.9)	285 (26.0)	26 (22.8)	259 (26.4)	
	Obesity (≥30.0)	203 (18.5)	23 (20.2)	180 (18.3)	
Parity	P0	938 (82.7)	96 (79.3)	842 (83.1)	0.362
	P1+	196 (17.3)	25 (20.7)	171 (16.9)	
PCOS	No	1031 (90.8)	109 (90.1)	922 (90.9)	0.891
	Yes	104 (9.2)	12 (9.9)	92 (9.1)	
Endometriosis	No	1059 (93.3)	118 (97.5)	941 (92.8)	0.053
	Yes	76 (6.7)	3 (2.5)	73 (7.2)	
Medication	No	741 (65.3)	78 (64.5)	663 (65.4)	<0.001
	Non-Hormonal Medication	357 (31.5)	32 (26.4)	325 (32.1)	
	Hormonal Medication	37 (3.3)	11 (9.1)	26 (2.6)	
Medical History	No	628 (55.3)	64 (52.9)	564 (55.6)	0.636
	Yes	507 (44.7)	57 (47.1)	450 (44.4)	
Ovarian Surgery	No	1102 (97.1)	115 (95.0)	987 (97.3)	0.257
	Yes	33 (2.9)	6 (5.0)	27 (2.7)	
Tubal Surgery	No	1096 (96.6)	116 (95.9)	980 (96.6)	0.857
	Yes	39 (3.4)	5 (4.1)	34 (3.4)	
Smoking Status	No	1074 (94.6)	116 (95.9)	958 (94.5)	0.669
	Yes	61 (5.4)	5 (4.1)	56 (5.5)	
Occupation Category	Unemployed/Homemaker	72 (6.3)	0 (0.0)	72 (7.1)	NA
	Healthcare Workers (BOC 3)	203 (17.9)	76 (62.8)	127 (12.5)	
	First Responders	3 (0.3)	3 (2.5)	0 (0.0)	
	Professional/Managerial Roles (BOC 0, 1, 2, 4)	673 (59.3)	17 (14.0)	656 (64.7)	
	Trades/Manufacturing/Service (BOC 6, 7, 8, 9)	133 (11.7)	22 (18.2)	111 (10.9)	
	Other or Non-Traditional (BOC 5)	51 (4.5)	3 (2.5)	48 (4.7)	

Note: BMI = body mass index. PCOS = Polycystic Ovarian Syndrome. NA = not applicable. BOC = broad occupational categories [18]. Data expressed as *n* (%) or median [IQR].

**Table 2:** Median AMH levels and low AMH (<7 pmol/L) by working group.

Outcome Measure	Overall (*n* = 1135)	Daytime Work (*n* = 1014)	Shift Work (*n* = 121)			*p*-Value
AMH level (pmol/L), median [IQR]	17.20 [9.00, 30.00]	17.20 [9.10, 30.00]	17.10 [8.10, 31.00]			0.930
			Evening shift * (*n* = 12)	Night shift * (*n* = 7)	Rotating shift (*n* = 102)	
			20.12 [10.50, 41.00]	23.00 [13.30, 23.25]	17.05 [8.10, 30.75]	
Low AMH (<7 pmol/L), *n* (%)	217 (19.1)	190 (18.7)	27 (22.3)			0.500
			Evening shift * (*n* = 12)	Night shift * (*n* = 7)	Rotating shift (*n* = 102)	
			3 (25.0)	1 (14.3)	23 (22.5)	

* These estimates are unstable due to small sample size.

**Table 3:** Association between risk factors and AMH levels.

	Univariable Model	Multivariable Model	
Risk Factor	Percent Difference in AMH Level (95% CI)	Percent Difference in AMH Level (95% CI)	*p*-Value
Shift Work	−0.85% [−19.29%, 21.81%]	2.30% [−16.19%, 24.87%]	0.823
Partner Shift Work	3.78% [−9.96%, 19.61%]		
Age (years)	−7.70% [−9.30%, −6.07%]	−6.67% [−8.29%, −5.03%]	0.000
BMI Class Underweight	−38.10% [−55.81%, −13.19%]	−37.75% [−54.65%, −14.56%]	0.003
BMI Class Normal Weight	Reference	Reference	
BMI Class Overweight	−2.54% [−16.55%, 13.81%]	−6.77% [−19.52%, 7.98%]	0.349
BMI Class Obesity	−13.75% [−27.57%, 2.72%]	−17.34% [−30.06%, −2.29%]	0.026
PCOS	174.71% [121.99%, 239.96%]	155.03% [106.16%, 215.48%]	0.000
Endometriosis	−18.63% [−36.87%, 4.89%]		
Ovarian Surgery	−30.96% [−52.66%, 0.69%]		
Hormonal Medications	−20.36% [−44.29%, 13.85%]	−24.05% [−46.40%, 7.62%]	0.122

AMH = anti-Müllerian hormone. BMI = body mass index. PCOS = Polycystic Ovarian Syndrome. Percent difference = [exp(β) − 1] × 100; AMH was log-transformed due to its non-normal distribution; coefficients (β) were back-transformed and are expressed as percentage differences.

**Table 4:** (a) Univariable analysis for the association between risk factors and low AMH (<7.0 pmol/L); (b) multivariable analysis for the association between risk factors and low AMH (<7 pmol/L).

Risk Factors	Odds Ratio for Low AMH	95% CI	*p*-Value
**(a)**			
Shift Work	1.246	0.777, 1.94	0.345
Partner Shift Work	1.112	0.799, 1.534	0.525
Age (years)	1.189	1.131, 1.253	0.000
BMI Class Underweight	2.012	0.252, 1.04	0.051
BMI Class Normal Weight	Reference		
BMI Class Overweight	0.718	0.356, 1.53	0.370
BMI Class Obesity	0.634	0.305, 1.383	0.234
PCOS	0.238	0.092, 0.506	0.001
Endometriosis	1.238	0.686, 2.123	0.457
Ovarian Surgery	2.175	1.001, 4.464	0.039
Hormonal Medications	1.594	0.725, 3.243	0.217
**(b)**			
Shift Work	1.139	0.683, 1.844	0.606
Age (years)	1.172	1.113, 1.237	0.000
BMI Class Underweight	2.019	0.946, 4.087	0.058
BMI Class Normal weight	Reference		
BMI Class Overweight	1.512	1.037, 2.196	0.030
BMI Class Obesity	1.272	0.819, 1.95	0.276
PCOS	0.276	0.105, 0.600	0.003
Non-Hormonal Medication	1.115	0.789, 1.568	0.533
Hormonal Medication	1.888	0.796, 4.135	0.126

AMH = anti-Müllerian hormone. BMI = body mass index. PCOS = Polycystic Ovarian Syndrome. CI = confidence interval.

## Data Availability

The datasets generated during and/or analyzed during the current study are available from the corresponding author on request.
